# Supplementary material for: The 2015-2016 El Niño increased infection parameters of copepods on Eastern Tropical Pacific dolphinfish populations
Source: PLoS One. 2020 May 11;15(5):e0232737. doi: 10.1371/journal.pone.0232737 (PMC7213719; doi:10.1371/journal.pone.0232737)
Supplement: S1 Appendix — (DOCX) [file pone.0232737.s001.docx]

**S1 Appendix. Oceanographic variables from ONI 1+2 region during December 2013 to November**

**2015.**

| Year | Month | Sea surface temperature (SST) | Oceanic Niño Index (ONI 1+2) | Salinity | Chlorophyll *a* |
| --- | --- | --- | --- | --- | --- |
| 2013 | December | 22.61 | 22.61 | 33.42 ± 0.62 | 1.01 ± 1.86 |
| 2014 | January | 24.79 | 24.79 | 32.96 ± 0.89 | 1.01 ± 0.14 |
| 2014 | February | 25.40 | 25.40 | 33.12 ± 0.89 | 1.11 ± 3.61 |
| 2014 | March | 25.86 | 25.86 | 33.91 ± 0.64 | 0.97 ± 3.7 |
| 2014 | April | 25.23 | 25.23 | 34.38 ± 0.41 | 1.37 ± 6.56 |
| 2014 | May | 25.57 | 25.57 | 33.87 ± 0.97 | 1.16 ± 3.58 |
| 2014 | June | 24.51 | 24.51 | 33.25 ± 1.12 | 0.61 ± 0.7 |
| 2014 | July | 22.98 | 22.98 | 33.40 ± 0.85 | 0.65 ± 1.98 |
| 2014 | August | 21.91 | 21.91 | 33.39 ± 0.72 | 0.61 ± 2.17 |
| 2014 | September | 21.30 | 21.30 | 33.14 ± 0.74 | 0.71 ± 1.68 |
| 2014 | October | 21.54 | 21.54 | 32.88 ± 0.68 | 0.49 ± 1.40 |
| 2014 | November | 22.53 | 22.33 | 32.70 ± 1.01 | 0.63 ± 1.49 |
| 2014 | December | 22.90 | 22.90 | 32.79 ± 0.86 | 0.58 ± 1.08 |
| 2015 | January | 24.13 | 24.13 | 32.76 ± 0.97 | 0.53 ± 1.14 |
| 2015 | February | 25.59 | 25.59 | 33.39 ± 0.95 | 0.98 ± 4.33 |
| 2015 | March | 26.69 | 26.69 | 34.25 ± 0.82 | 1.27 ± 3.68 |
| 2015 | April | 26.95 | 26.95 | 34.64 ± 0.51 | 1.11 ± 5.62 |
| 2015 | May | 26.71 | 26.71 | 34.27 ± 0.70 | 0.95 ± 3.46 |
| 2015 | June | 25.42 | 25.42 | 34.16 ± 0.81 | 1.03 ± 2.23 |
| 2015 | July | 24.48 | 24.48 | 34.45 ± 0.68 | 0.89 ± 2.25 |
| 2015 | August | 22.88 | 22.88 | 34.22 ± 0.57 | 0.95 ± 1.43 |
| 2015 | September | 22.91 | 22.91 | 33.98 ± 0.89 | 0.81 ± 1.09 |
| 2015 | October | 23.31 | 23.31 | 33.52 ± 0.73 | 0.58 ± 0.77 |
| 2015 | November | 23.83 | 23.83 | 33.16 ± 0.56 | 0.49 ± 0.87 |
