# Supplementary material for: The 2015-2016 El Niño increased infection parameters of copepods on Eastern Tropical Pacific dolphinfish populations
Source: PLoS One. 2020 May 11;15(5):e0232737. doi: 10.1371/journal.pone.0232737 (PMC7213719; doi:10.1371/journal.pone.0232737)
Supplement: S2 Appendix — (DOCX) [file pone.0232737.s002.docx]

**S2 Appendix.** Average SST in the Niño (1+ 2) region, base periods of 30 years. <http://www.cpc.ncep.noaa.gov/products/analysis_monitoring/ensostuff/ONI_change.shtml>
