# Supplementary material for: The 2015-2016 El Niño increased infection parameters of copepods on Eastern Tropical Pacific dolphinfish populations
Source: PLoS One. 2020 May 11;15(5):e0232737. doi: 10.1371/journal.pone.0232737 (PMC7213719; doi:10.1371/journal.pone.0232737)
Supplement: Attachment — Submitted filename: second review of dolphinfish EN parasites.pdf [file pone.0232737.s004.pdf]

Review of "*The 2015-16 El Niño increased infection parameters of copepods on Eastern Tropical Pacific dolphinfish populations*"

Santana-Piñeros et al present a revised version of their manuscript relating the parasitic copepod load on dolphinfish caught off the coast of Ecuador to regional oceanographic conditions, specifically an increase in parasitism during the recent strong El Niño event.

General comments

This remains an interesting manuscript connecting a regional warming event to parasite loads in an important fishery species, making projections regarding effects of long-term climate change on the same. However, it also remains fraught with errors in English spelling and grammar, and fails to consider alternative hypotheses or the limits of the correlative evidence presented for mechanistic inference. For example, can you explore other potential hypotheses regarding the mechanism of host-driven increases in parasite loads during the dry season, when hosts were smaller? Maybe they are less well fed and thus weaker in responding to parasites, or they are migrating from a different water mass during the dry season – maybe even representing a different host population? If you are consistently seeing smaller dolphinfish during the dry season I find it more likely that you are seeing different fish during this period of the year than that you are seeing the same population in both seasons and that these individuals are becoming smaller and then bigger again on a yearly basis. In addition, the seasonal pattern (dry/cold vs wet/warm) is very similar to the LN-EN difference in terms of water temperature, dolphinfish size, and infection parameters. This suggests similar driving mechanisms.

Specific comments

Line 38: modified by El Niño, affecting several ecological processes. Parasites and other marine organisms respond to environmental variation, but the influence…

Line 67: (and throughout) During El Niño (EN) events *(no "the" before El or ENSO)*

Line 75: ~~performance~~

Line 121: both temperature ranges should be in low-high order, and you cannot cite a website directly in manuscript text (please see Plos One literature citation guidelines)

Line 194: should this be a two-way ANOVA with season and EN as crossed or nested factors?

Line 246: different (*not difference*)

Line 333: This is the first tropical survey to provide evidence that the EN period promotes…

Line 342: widely *(not profusely)*

Line 348: Sentence needs to be re-written, whole document checked for basic English

Line 351: although it prefers an SST range of 21-30 C

Line 360: Sentence needs to be re-written

Line 375: missing a period

Line 376: an historical event

Line 385: My experience seeing fish in the water has been that larger animals tend to have far more ectoparasites than smaller individuals.

Line 416: Sentence needs to be re-written

Line 420: temperature on the ~~copepod~~ reproductive parameters of copepods; also this sentence is a run-on, with changes in tense that need to be corrected

Line 454: ~~Apparently~~ ; more frequent in the ??? no citation? And next line cites a website? Too many formatting, stylistic, and grammatical errors.
