# Supplementary material for: The 2015-2016 El Niño increased infection parameters of copepods on Eastern Tropical Pacific dolphinfish populations
Source: PLoS One. 2020 May 11;15(5):e0232737. doi: 10.1371/journal.pone.0232737 (PMC7213719; doi:10.1371/journal.pone.0232737)
Supplement: Attachment — Submitted filename: Response to reviwers.docx [file pone.0232737.s003.docx]

Reply to referees’ comments. Each query is followed by an answer(A).

**General comments:**

1. Is the manuscript technically sound, and do the data support the conclusions?

The manuscript must describe a technically sound piece of scientific research with data that supports the conclusions.

Experiments must have been conducted rigorously, with appropriate controls, replication, and sample sizes. The conclusions must be drawn appropriately based on the data presented.

A= Thank you for your comments, which have contributed to improve the manuscript. In this new version we have supported the conclusions.

1) We have made a generalized additive model in order to determine if the El Niño event modifies the infection parameters.
2) We use “EN” instead of “SST”, since the infection parameters changed due to a set of variables that are modified by the EN event.
3) We included new information on the egg size and total length of copepods of the non-EN and the EN samples, in order to support the results of the manuscript.

We did not carry out a laboratory experiment, since dolphinfish is a major pelagic fish and its capture is rather difficult. Additionally, the contribution of this work consists in the effect of the EN event on parasitic copepods of wild fish populations. There are several studies of a few species of parasitic copepods vs SST or salinity, mostly under laboratory conditions that show an effect of these variables on the biology of the species. Most of these studies have been carried out in Chile or Norway (temperate zone), mainly due to the effect these species have on salmon aquaculture.

To date, no systematic studies (2 years) have been carried out in the study area, so we consider it to be new information not only for the biology of the fish and parasite species, but also in other effects of the EN events on the Tropical Eastern Pacific.

2. Has the statistical analysis been performed appropriately and rigorously?

Reviewer #1: Yes

Reviewer #2: I Don't Know

Reviewer #3: I Don't Know

A= We have included an archive of supplementary data to clarify doubts about the analyzes. In the same way, we did the analysis suggested by one of the reviewers (General Additive models or GAMLSS), which substantially helped to improve the manuscript and to modify our conclusions.

3. Have the authors made all data underlying the findings in their manuscript fully available?

Reviewer #1: Yes

Reviewer #2: Yes

Reviewer #3: Yes

A= Anyway, we have included a supplementary data file.

4. Is the manuscript presented in an intelligible fashion and written in standard English?

PLOS ONE does not copyedit accepted manuscripts, so the language in submitted articles must be clear, correct,

and unambiguous. Any typographical or grammatical errors should be corrected at revision, so please note any

specific errors here.

Reviewer #1: Yes

Reviewer #2: Yes

Reviewer #3: No

A= The paper has been professionally proofread by an English native speaker before submission.

**Reviewer #1:**

Santana-Pineros et al present a relevant study showing heightened prevalence and infection load of ectoparasitic copepods on dolphinfish during the recent severe El Nino in the TEP. I think the manuscript could use a careful edit by a native English speaker, I tried to assist as much as I could with grammar and spelling.

A= Manuscript has been revised by an English native speaker.

Also data availability statement is made in the submission form, but there is no text in methods or elsewhere specifying where and how one could obtain raw data. Also mentioned are supplemental files for data and maybe some methods? Results? Yet no supplemental files were in the pdf for review, nor are they cited anywhere in the text.

A= Supplemental files were included.

The abstract needs to include specific values from the results section, not just increase/decrease statements.

A= Done. Line 57-59.

Finally, some restraint is warranted in extrapolating the data observed to draw general conclusions. While there was a clear correlation between parasite infection rates and El Nino, without proper controls or comparisons across species or multiple El Nino events it is difficult to attribute causation. For instance, in Figure 3A, it would appear to me that C. bonito prevalence is highly seasonal, rather than being directly driven by the El Nino event. Since study is specific to TEP, maybe restrain intro to TEP

A= We fully agree. Results indicate that parasites and environmental variables have a 11 or 12 months’ annual pattern, respectively. However, this pattern is broken during the EN event. In this new version we explain these arguments.

To support this information, we include a GAMLSS analysis. This analysis detected that infection parameters are explained by various environmental variables and EN. In this sense, we now use EN instead of SST.

Remarks on lines 41, 46, 47, 52, 77, 80, 86, 88, 104, 109, 121, 160, 171, 185, 227, 229, 233, 239, 241, 243, 253, 259, 264, 267, 281, 305, 324, 338, 357, 361, 365, 371, 388 were changed it as suggested.

Line 45: ... in the dolphinfish Coryphaena hippurus and oceanography during the 2015-16 El Nino event. *** ENSO is not synonymous with El Nino, which is just one state or extreme of the ENSO cycle (which also includes La Nina on the opposite side of the spectrum). When referring to this event, please use El Nino or EN, not ENSO.

A= Done. We used El Niño (EN) instead of ENSO throughout the manuscript.

Line 55: both copepod species and increasing SST.

A= This phrase was changed by “We also observed a positive correlation between the increasing SST values and the infection parameters of both copepod species”

Line 57: Please state actual values by which prevalence and intensity of C. bonito and Ch. Quaternia increased. Also is this truly the only study that has looked at parasitic copepod abundance relative to sea surface temperature variation? Statements such as this throughout the MS should be carefully double-checked or re-worded.

A= Increased values of prevalence and mean intensity for both copepod species were included, as well as “EN” was used instead of “climate variability”. We have double check and we did not find any other study focused on the relationship of EN and infection parameters of parasite copepods worldwide. Therefore, we have maintained our statement.

Line 64: El Nino-Southern Oscillation (ENSO) events create fluctuations in sea surface temperature (SST) >0.5 􀀀C in the Tropical Eastern Pacific (TEP). During El Nino events,…

A= This phrase was changed by “The El Niño-Southern Oscillation (ENSO) creates fluctuations in sea surface temperature (SST) of the Tropical Eastern Pacific (TEP), and it has been suggested that its frequency and magnitude will increase in the future.”

Line 68: waters have a profound influence on local weather, ocean conditions, and marine and terrestrial ecosystems.

A= Sentence was changed.

Line 69: Consider moving sentence on line 75 that starts “it has been suggested…” to before this sentence, since it provides precedent for climate change connection (having not been mentioned before this point leaves the reader bewildered).

A= Sentence was changed.

Line 98: populations could be better managed in the region…

A= Sentence was changed.

Line 113: SOI is listed here without previous mention or acronym definition; needs explaining.

A= The paragraph was changed. “SOI” is not mentioned in the new manuscript.

Line 114: we used a 2-year time series of monthly sea surface temperature…

A= The paragraph was changed.

Line 116: With prevalence and intensity of parasites in dolphinfish…

A= The paragraph was changed.

Line 117: Tropical Eastern Pacific during the El Nino event of 2015-2016.

A= The paragraph was changed.

Line 148: To calculate the sample size, we calculated the prevalence of both C. bonito and Ch. Quaternia in 154 individual hosts during pre-El Nino years. (Is this correct? Please be more detailed and specific how/why these fish were used as a baseline. What were the oceanographic conditions during the pre-collection phase relative to EN year?

A= Done. We improved writing and add more detailed and specific information on how/why these fish were used as a baseline.

Line 153: minimum monthly sample size to achieve what? Detect a single infection? Again please be specific.

A= We improve writing and add more details.

Line 186: with monthly values of SST, salinity…

A= Sentence was changed.

Line 211: Did you measure length, weight? Can you look at length/weight ratios and whether they varied between non– EN and EN periods, or any other parameter of body condition or physical health that could otherwise show an overall health effect of EN and/or copepod infection?

A= This is a very good observation. Unfortunately, we only measured the length of host because we only have access to fish organisms once they were eviscerated and filleted. We have included this information in "Parasitological examination" (Lines 149-150). However, we did an analysis to determine if the size of eggs, total length of females or sex proportion of copepods varied between the non-En and EN periods.

Line 216: ENSO periods do not affect host sizes or the size distributions of dolphinfish populations.

A= The paragraph was changed.

Line 224: SST increased steadily from February 2015 until the end of the study, while..

A= The paragraph was changed.

Line 245: mean intensity and SOI 1+2 with no lag. …a shift of SST variability related to the influence of SOI 1+2 coincident with changes in Chl-a and salinity, all of which affected the infection parameters of… NOTE: since these

oceanographic factors co-varied you cannot partition the effects of each in this MS.

A= Agree. We re-phrase it.

Line 267: Since SST, Chla-a, and salinity co-varied over the study period, they may have acted simultaneously to trigger increased infection levels of Ch. Quaternia on C. hippurus. Since these oceanographic variables are highly correlated, it was impossible to detect the effect of each factor alone. … intensity and SOI 1+2 with a 4-month lag.

A= Agree. We re-phrase it.

Line 278: SST (C), chlorophyll a (Chl-a)… and the prevalence and infection intensity…

A= Sentence was eraser.

Line 287: A specific case where extrapolation goes to far; correlation between ENSO and copepod infection prevalence does not signify causation.

A= We included other information to support our conclusions.

Line 289: Although we quantified seasonal and annual patterns of several oceanographic variables, only SST… was associated with the El Nino event of 2015-2016… average temperature relative to a 136-year record… SST for the

global ocean was… Ocean temperatures during the first three months of 2015 were each the third warmest on record for their respective months.

A= We changed several paragraphs of the discussion and included sections explaining the environmental, host and parasite dynamics. The environmental variability is discussed in lines 355-376.

Line 307: parasitic copepod species might respond synergistically to two events: the increase in the number of host individuals *NOTE: this is the first mention of increased population size during El Nino, it is not reported on or shown in figures or tables in the Results section. Or are you referring to the citation on line 317? If so that citation should be mentioned before this statement.

A= In this new version we include a section of the “host factor”, lines 337-354. We have redesigned all the figures, including a figure (Fig 1) of total length and included citations to support our results.

Line 312: This sentence is almost illegible and I wouldn’t try to rewrite since I am unsure of the context. For this reason and myriad other small edits needed, I would again recommend the MS be carefully proof-read by a native English speaker.

A= We have rewritten the paragraph to make it more legible please see lines 361-365. Additionally, the new version has been professionally proofread by an English native speaker before submission.

Line 320: We documented … copepod species and SST. Our observations suggest that.. an increase in the prevalence and mean intensity…

A= This paragraph was changed.

Line 327: Rising infection parameters could be related to the higher number of suitable hosts due to high temperature *NOTE: again tenuous since you did not show data to support this…rate of infection between hosts, or increased reproduction rates of parasites due to high temperature.

A= We have explained this argument with data derived from our results, please see lines 417-433.

Line 332: parasite infection is host fish population… copepod parasites, proximity…

A= This paragraph was changed.

Line 343: four months, in contrast to mean intensity, which showed an immediate response… triggers an increase in the… per infected host, which… * NOTE: Any evidence for Ch. quaternia to be in competition for space on hosts? Please cite authority on this statement.

A= Citation has been included, lines 445-451.

Line 349: Additionally, this lag (up to 4 months).

A= This sentence was eraser

Figure 1 Decimal values should be labelled with a period, not a comma.

A= Done.

**Reviewer #2:** Copepods are not convincingly presented to be good bioindicators.

The sampling number in the el nino period is less than the non el nino period.

The discussion is presented speculatively and the presentation of information is not in a logical sequence. The main conclusion of the manuscript is that the increase in temperature in the el nino causes an increase in the copepod population, but in the graph it is verified the increase in temperature gradually, in other words, the peaks in the el nino do not seem significant to affirm that they influence the copepod population.

A= In last years, it has been observed that global ecological changes can directly influence the infection dynamics of parasites (Burge et al., 2014), mainly in those with direct life cycle (crustaceans, copepods and monogeneans) (Koprivnikar et al., 2010). Various investigations have demonstrated that the parasitic copepods quickly spread on the fish hosts in warm water and the high water temperature promotes faster hatching of eggs (Hayward et al., [2009](https://link.springer.com/article/10.1007/s00436-011-2724-6#CR27); Johnson et al., [2004](https://link.springer.com/article/10.1007/s00436-011-2724-6#CR31)). Hakalahti and Valtonen (2003) observed that the generations of ectoparasites increased with the variation in the water temperature in *Salmon trutta* farms in Finland. Additionally, temperature also influences the immune system in fishes and might affect intensities of parasites indirectly (Johnson et al., 2004). Warmer temperatures increased the metabolic rate and reduced the reproduction time of ectoparasites and this is the principal environmental factor in the development and transmission of ectoparasites.

**Burge, C. A., C. M. Eakin, C. S. Friedman, Brett - Froelich, K. P. Hershberger, E. E. Hofmann, L. E. Petes, K. C. Prager, E. W. Bette, L. Willis, S. E. Ford, and C. Drew Harvell**. (2014). Climate Change Inﬂuences on Marine Infectious Diseases: Implications for Management and Society. Annual review of marine science 6: 111-129.

**Koprivnikar, J., Lim, D., Fu, C. and Brack, S. H. M.** (2010). Effects of temperature, salinity, and pH on the survival and activity of marine cercariae. *Parasitology Research*. doi: 10.1007/s00436-010-1779-0.

**Hakalahti, T., E. T. Valtonen, and Y. Lankinen.** (2004). Efficacy of Emamectin benzoate in the control of *Argulus coregoni* (Crustacea: Branchiura) On rainbow trout *Oncorhynchus mykiss*. Diseases of aquatic organisms 60: 197-204.

**Hayward CJ, Bott NJ, Nowak BF.** (2009). Seasonal epizootics of sea lice, *Caligus* spp., on southern Bluefin tuna, *Thunnus maccoyii* (Castelnau), in a long-term farming trial. J Fish Dis 32:101–106

**Johnson SC, Treasurer JW, Bravo S, Nagasawa K, Kabata Z.** (2004). A review of the impact of parasitic copepods on marine aquaculture. Zool Stud 43:229–243

**Reviewer #3:** Dear Authors,

I have been asked to review your manuscript ‘El Nino increased infection parameters of copepods on Eastern Tropical Pacific dolphinfish populations’ (PONE-D-19-16590). The idea of testing the response of parasites to climate variability is very interesting, and I appreciate the amount of work you have put in the study. However, I have some major concerns (and a few minor comments) that first need to be solved before this manuscript can be accepted for publication.

First of all, I am afraid that your conclusion is not entirely supported by your analyses. You conclude that tropical parasites are sensitive to climate variability. However, you support your conclusion entirely correlative ‘evidence’. When there is a correlation between two variables (like SST and infection parameters) it does not necessarily mean that the sea surface temperature is responsible for increases in infections. It would have been better to analyze this with general additive models so you have a better idea of causality.

A= We fully agree with referee´s comments and suggestions, so we have incorporated a GAMLSS analysis in order to have a stronger support for our hypothesis, results showed that fluctuations on prevalence and mean intensity could be explained by different oceanographic (SST, Chl-*a*, ONI 1+2) and biological variables (TL) affected by the EN event. The sustained increases in SST and ONI 1+2 modified the infection parameters and other environmental variables, mainly in the dry season during the EN, altering the seasonal cycle (dry-rainy). These results are explained in new version.

Furthermore, it would be good for the manuscript to see some additional prove. For example, an experiment that shows that infective larvae have higher development rates with higher temperatures. There has been some speculation about this in the discussion (for other copepod species), but a small experiment would give the reader more confidence in your conclusion.

A= As above mentioned, we do not carry out a laboratory experiment, since dolphinfish is a major pelagic fish and its capture is rather difficult. Additionally, we do not have an experimental marine fish laboratory. Fortunately, we have all copepods collected during 2 years and we decided to measure the size of eggs and females to give more confidence to our results. Please refer to lines 318-323; 416-433 and Figure 5.

In the discussion there is also speculation about an increase in host abundance as a result of higher temperatures. Are there not data (fish landings for example) to support this idea? In fairness, the discussion that should support your conclusions is way to speculative for the reader to believe that your conclusions are true and what the prime cause of your observations could be.

A= We have incorporated analysis of the host´s TL during the non-EN and EN periods (Lines 236-249 and Fig. 1) and include a section in discussion on the temporality of dolphinfish during the non-EN and EN periods (Lines 337-354).

Second, the English of the manuscript is not good enough for publication. I highly recommend giving your manuscript to a native English speaker before submitting it again. In the minor comments section I will give some examples of sentences that were unclear to me. Here and there I have found also some Spanish words.

A= Manuscript was revised by an English native speaker

Third, the figures are too crowded. Having four different y-axes instead of one is very confusing. Also, Figure 2D, 3E and 4D require more explanation.

A= All figures were edited. New figures have only two y-axes. For example; figure 2 show temporal fluctuation of environmental variables, each variable (SST, Chl-a and salinity) was plotted with ONI, and only spectral density was done. We believe that this new version is easier to understand.

MINOR COMMENTS

L69: Climate change or ENSO?

A= El Niño-Southern Oscillation (ENSO) includes El Niño and La Niña. We only observed El Niño, so we now use ““El Niño (EN) or “non-El Niño (non-EN)” instead of “ENSO”

Remarks on lines 75/76, 77, 78, 78/79, 80, 83-85, 120, 126, 148, 161, 165, 185-187, 193, 222, Fig. 2, 322, 327, were changed it as suggested.

L86: Substitute “load has long been recognized as a factor affecting” by “are known to affect”.

A= Sentence was changed.

L89: Substitute “leave” by “thereby leaving”. Substitute “exposing the host to” by “that can result in”

A= Sentence was changed.

L113: This is the first time you use the abbreviations, please write out.

A= The paragraph was changed. Our objective was adjusted to evaluate the infection parameters between the El Niño and the non El Niño. All abbreviated words were declared the first time they are mentioned in the document.

L115/116: This is the second time you use abbreviations, please do not write out.

A= Sentence was modified.

L116: What exactly is the Southern Oscillation index? Please explain.

A= We included explanation in lines 169-180.

L127_134: How are the fish caught? With nets? Long lines? Is there a chance that the parasites are removed by the fishing method?

A= We have included a sentence in “sampling” section (lines 133-134).

L146: How many fish per month?

A= We have incorporated more details, lines 136-138.

L147/148: Why are the lengths of the ENSO and non-ENSO period different?

A= The length of the ENSO and non-ENSO periods are different because when the research was planned, we did not know it would be an ENSO period. Unfortunately, although the ENSO period began in April 2015, the samplings could not extend beyond what was planned (December 2015) due to budget limitations and administrative decisions of the financial entity.

L148-155: This is very unclear to me. I had to read reference 14 to understand what you did. Please be more detailed here.

A= We improve writing and incorporated more details (lines 138-147).

L164: Increasing concentrations of glycerol. Please be more specific.

A= We incorporated more details (line 156).

L175/176: I really do not know what you mean with these values and regions, and why they are important for your study.

A= The new version makes clear the use of these variables. It helped to explain the environmental changes of the EN and its influence on the infection parameters.

L178/178/182: The websites are not working, please update the links

A= We update the links.

L190: Single or Singular?

A= “Spectral analyses” instead of “single” (line 214).

L190-194: The part “for data with equally spaced sampling points in time” does not fit with the rest of the sentence. Please rephrase..

A= Sentence was rephrase line 217-220.

L215: Substitute “followed the same trend” with “was the same”.

A= Paragraph was changed, lines 236-249.

L216: Substitute “does” with “do”, “sizes” with “size”, “or” with “and”.

A= Paragraph was changed.

L217: Dolphinfish populations.

A= Sentence was erased.

Fig 1: Please add unit to x-axis.

A= Done.

L222: What do the numbers between the barracks mean?

A= We include this information in methodology lines 235-236.

L243: What do you mean with lag 0? Please explain.

A= Lag 0 means an immediate response of a dependent variable due to modification of independent variable. You can find definition of lag in M&M, lines 228-229.

Fig 3A: How can the prevalence be higher than 100%? Please check your data.

A= Prevalence was not higher than 100%, these is a visual effect. Figure 3 was modified to eliminate the visual effect.

L296: “Each third warmest”. What do you mean?

A= Writing was improved to make it clear, lines 375-377.

L298: Add “resulting in” after “year”. Remove “evolved”.

A= Sentences was changed.

L307-312: Speculative. What proof do you have?

A= We changed writing to avoid speculation, lines 378-416.

L312: “The current weakens system are often described” What do you mean?

A= This phrase was changed, lines 362-366.

L321: Replace “occurred” with “was observed”.

A= This sentence was eraser.

L327: Substitute “Infection parameters rising” with “Higher infection levels”.

A= This sentence was eraser.

L328: Substitute “the high temperatures” by “higher temperatures”

A= This sentence was eraser.

L328: How much evidence do you have for this? Please give some sources.

A= We provide evidence of changes in some reproductive parameters of C. bonito and the EN (lines 418-434).

L331: So do you mean these are favorable temperatures or not? Please draw a conclusion here.

A= This paragraph was eliminated.

L338-341: More a hypothesis than a conclusion how it is formulated here.

A= We changed the sentence to formulated a conclusion here.

L342-355: This does still not explain the lag phase between increasing intensity and prevalence. Do fish that are already infected become more infected?

A= This sentence was restructured and new arguments were included.
