# Supplementary material for: The 2015-2016 El Niño increased infection parameters of copepods on Eastern Tropical Pacific dolphinfish populations
Source: PLoS One. 2020 May 11;15(5):e0232737. doi: 10.1371/journal.pone.0232737 (PMC7213719; doi:10.1371/journal.pone.0232737)
Supplement: Attachment — Submitted filename: Response to reviwers.docx [file pone.0232737.s005.docx]

Reply to referees’ comments. Each query is followed by an answer(A).

**General comments:**

This remains an interesting manuscript connecting a regional warming event to parasite loads in an important fishery species, making projections regarding effects of long-term climate change on the same. However, it also remains fraught with errors in English spelling and grammar, and fails to consider alternative hypotheses or the limits of the correlative evidence presented for mechanistic inference. For example, can you explore other potential hypotheses regarding the mechanism of host-driven increases in parasite loads during the dry season, when

hosts were smaller? Maybe they are less well fed and thus weaker in responding to parasites, or they are migrating from a different water mass during the dry season – maybe even representing a different host population? If you are consistently seeing smaller dolphinfish during the dry season I find it more likely that you are seeing different fish during this period of the year than that you are seeing the same population in both seasons and that these individuals are becoming smaller and then bigger again on a yearly basis. In addition, the seasonal pattern (dry/cold vs wet/warm) is very similar to the LN-EN difference in terms of water temperature, dolphinfish size, and infection parameters. This suggests similar driving mechanisms.

A= Thank you for your comments, which have contributed to improve the manuscript. In this new version we have include other potential hypotheses for explained our result. These changes can be observed in the section “infection parameters annual patterns”. Additionally, the paper has been professionally proofread by an English native speaker before submission

I hope that this new version will be good enough to be considered for publication in the journal*.*

**Specific comments**

Line 38: modified by El Niño, affecting several ecological processes. Parasites and other marine organisms respond to environmental variation, but the influence…

A = Done

Line 67: (and throughout) During El Niño (EN) events (no “the” before El or ENSO)

A = Done

Line 75: performance

A = Done

Line 121: both temperature ranges should be in low-high order, and you cannot cite a website directly in manuscript text (please see Plos One literature citation guidelines)

A= Done

Line 194: should this be a two-way ANOVA with season and EN as crossed or nested factors?

A = Changed “one” by “two”

Line 246: different (not difference)

A= Done

Line 333: This is the first tropical survey to provide evidence that the EN period promotes…

A = Done

Line 342: widely (not profusely)

A = Done

Line 348: Sentence needs to be re-written, whole document checked for basic English

A = We re-written sentence. Manuscript has been revised by an English native speaker

Line 351: although it prefers an SST range of 21-30 C

A= Done

Line 360: Sentence needs to be re-written

A= We re-written sentence

Line 375: missing a period

A= This sentence was eraser

Line 376: an historical event

A= Done

Line 385: My experience seeing fish in the water has been that larger animals tend to have far more ectoparasites than smaller individuals.

A= We explained this result in line 383 to 395

Line 416: Sentence needs to be re-written

A= We re-written sentence

Line 420: temperature on the copepod reproductive parameters of copepods; also this sentence is a run-on, with changes in tense that need to be corrected

A= We corrected this sentence

Line 454: Apparently; more frequent in the ??? no citation? And next line cites a website? Too many formatting, stylistic, and grammatical errors.

A= We corrected this sentence
