# Supplementary material for: The 2015-2016 El Niño increased infection parameters of copepods on Eastern Tropical Pacific dolphinfish populations
Source: PLoS One. 2020 May 11;15(5):e0232737. doi: 10.1371/journal.pone.0232737 (PMC7213719; doi:10.1371/journal.pone.0232737)
Supplement: Attachment — Submitted filename: Response to reviwers.docx [file pone.0232737.s007.docx]

Reply to referees’ comments. Each query is followed by an answer (A).

**General comments:**

Santana-Piñeros et al. have submitted their revised version of a study investigating the effects of the 2015-16 El Niño event on ectoparasite loads of dolphinfish. The manuscript has been thoroughly revised and most of the grammatical/English issues have been corrected (a few minor changes still needed, see specific comments). In addition, the authors have modified their language to consider a more nuanced explanation of the environmental factors driving variation in ectoparasite infection rates, in light of the relatively short sampling window around the major EN event.

For the most part I feel that our issues raised have been dealt with. However, there are still some inconsistencies/shortcomings in the interpretation of the results. For instance, the bulk of the article (including the title!) suggest that EN increased infection parameters of copepods on dolphinfish, yet in the discussion (see lines 420-421), you state that infection rates of *C. bonito* were actually higher during the non-EN period.

A= Thank you for your comments, which have substantiallyt improved the manuscript.

We have reviewed the statiscal analyzes presented in the previous version of the manuscript and detected a mistake in the SST values, which were for the 3.4 region and not 1+2 region. We have corrected it and the generalized models showed a higher explained deviance and even the prevalence of *C. quaternia* could be explained. Our results confirmed that the increase in SST has risen the infection parameters of the two parasite copepods (see Figs. 3D, 4D). In this new version, we re-written the paragraph (lines 427-439) and we have included a figure in a supplementary file.

In addition, you state that mean host TL did not differ between EN and non-EN periods, but then in the discussion (e.g., line 427) you use increased TL during EN as an explanation for decrease in *C. bonito* infection during EN. If you truly think that host size was an important factor interacting with the oceanographic variables to predict infection rates, you might consider analyzing different size classes separately.

A= This argument was removed from the manuscript. We separately analyzed different size classes and there were no differences in prevalence with respect to the previous analyzes.

Finally, I have some doubts about the spectral analysis methodology. Since this is a relatively short window of time (24 months), a peak spectral density of 11 months is nothing more than saying that one year was different from the next. This alludes to my original concern with this manuscript, that the short sampling period is insufficient to establish an EN effect per se, but rather a difference between years that might be explained by oceanographic parameters. For this reason I wonder if ONI is really an informative explanatory variable or if you should simply focus on SST, salinity, etc.

A= Spectral analysis methodology does not allow comparison between years, but repeated patterns over time. The longer the time series, the different time patterns can be detected. Due to our short time series, the spectral analysis detected that every 11 months the environmental variability in the study area is repeated, which is consistent with the oceanographic temporality in that area. The difference between years was given by the SST, with higher values in 2015 (Fig. 2). In this sense, we rewrote the paragraph taking the reviewers' suggestion, focusing on explaining the results with SST and not ONI.

**Specific comments**

Line 39: the influence of the EN cycle… (“the” is placed before EN in this case)

A= Done.

Line 44: since the term “TEP” does not appear later in the abstract, no need to define acronym here (just say Tropical Eastern Pacific). Acronym is correctly defined in the main body of the text on line 65.

A= Done.

Line 51: Consider reporting specific number of degrees C SST increase here (rather than just “SST increased consistently”)

A= We add information in abstract (Lines 48-56) and include a specific value of SST (Line 57).

Line 52: “The SST increase interrupted the seasonal patterns of salinity and Chl-a. It is suggested that EN had the greatest influence on the infection parameters of both copepod species.” -- These two sentences do not make sense and do not add information to the abstract. You state in the previous line that only SST increased over the EN period, and the entire paper is about the influence of EN on infection parameters of copepods. I recommend removing these two sentences and beginning next sentence with simply: “We observed…”

A= We deleted those lines and modified part of the abstract, showing the most important results of the manuscript.

Line 64: Indent first sentence of each paragraph (see throughout MS), and in this case it is correct to start with “The El Niño…”

A= Done.

Line 74: “Affects performance**,** population dynamics…”

A= Done.

Line 100: Risso, 1810 is placed in parentheses while other citations of authorities who named species were not in parentheses; please be consistent

A= International Code of Zoological Nomenclature (ICZN) establishes: In citing the name of an author, the surname is given in full, not abbreviated. The date of publication in which the name was established is added for example *Mugil curema* Valenciennes, 1836. However, if species was described with other genera, the author and year are set in parentheses. For example, *Katsuwonus pelamis* (Linnaeus, 1758) was described as *Scomber pelamis* Linnaeus, 1758. Since the taxonomic status of *A*. *rochei* changed, then it is mandatory to have the surname in parenthesis.

Line 119: SST was defined earlier, can simply use acronym throughout manuscript

A= Done.

Line 135: … sampling of 154 dolphinfish to establish a baseline prevalence of infection during the non-EN period

A= Done.

Line 156: using specific literature taxonomic keys

A= Done.

Line 170: the EN conditions

A= Done.

Line 174: Values of the SST

A= Done.

Line 238: (mean 72.88 ± 12.45 SD) this is to define parameters upon first usage, can leave following values as numbers without units

A= Done.

Line 271: Please define UPS acronym upon first usage (for salinity)

A= Done.

Line 272: Please state which season and/or EN/Non-EN periods had greater/lower values for each of the environmental parameters measured (rather than just stating that they were significantly different)

A= We changed paragraph.

Line 291: 29% - 100%

A= Done.

Line 330: altering both environmental physical and biological oceanography

A= Done.

Line 341: Results (mean? TL in dry and rainy seasons) should be presented in the Results section

A= We delete this phrase.

Line 345: Can you discuss the approximate ages associated with these sizes? Are you suggesting these fish are being born and growing to maturity within a single season? Or are larger individuals migrating into these waters during the rainy season?

A= We cannot associate size classes with ages. We re-wrote this paragraph.

Line 358: I have a hard time interpreting the 11-month cycles statement. With only 24 months of data, you can really only say that one year was different from the next.

A= The time series detects patterns over time. In this sense, we do not say that one year is different from other, but that the variability of our oceanographic variables is repeated in 11-month cycles. These cycles are consistent with the dynamics of currents and seasonal temporality in the area.

Line 360: varies varied

A= Done.

Line 398: hosts

A= Done.

Line 407: dynamics of the parasites

A= Done.

Line 418: these are different mean SST values reported as long-term averages for these months than those reported on line 120

A= We change SST by air temperature in line 120.

Line 420-421: This statement runs directly in opposition to the title of the paper

A= We modified this statement.

Line 465: density-dependence, intra- and inter-specific competition,

A= Done.

Line 469: The future ENSO events

A= Done.
